# Morphology of the obturator nerve, an anatomical study with emphasis on its clinical implications

**DOI:** 10.12688/f1000research.147000.1

**Published:** 2024-04-23

**Authors:** Latha V. Prabhu, Rajanigandha Vadgaonkar, Ashwin R. Rai, Vandana Blossom, Mangala M. Pai, B.V. Murlimanju

**Affiliations:** 1 Department of Anatomy, Kasturba Medical College, Mangalore, Manipal Academy of Higher Education, Manipal, Karnataka, India

**Keywords:** Lumbosacral Plexus; Obturator Nerve; Nerve Entrapments; Pelvic Region

## Abstract

**Background:**

The goal of this cadaveric cross sectional study was to analyse the branching pattern of the obturator nerve morphologically and to determine its dimensions in embalmed cadavers.

**Methods:**

In this cross sectional study, we examined 50 embalmed adult cadaveric lower limbs. Sex was not taken into consideration in the analysis; however, a side-based comparison was performed. The measurements were performed using a digital Vernier caliper.

**Results:**

The branching of obturator nerve was observed at the pelvic cavity in 28 specimens (56%) and inside the obturator canal in 12 specimens (24%). The division of obturator nerve wasn’t observed in 10 specimens (20%). The length, width and thickness of the trunk of obturator nerve was 108.26 ± 9.53 mm, 2.84 ± 0.88 mm and 1.11 ± 0.35 mm. The width and thickness of the anterior and posterior divisions of obturator nerve measured 2.19 ± 0.82 mm, 0.9 ± 0.1 mm, 0.99 ± 0.6 mm and 0.71 ± 0.26 mm. The topography of branching of obturator nerve from the superior and inferior border of the obturator foramen was located at 1.48 ± 0.58 mm and 3.07 ± 1.1 mm away. The length of anterior division of the obturator nerve measured 110.88 ± 12.02 mm over the right side and 107.13 ± 7.81 mm over the left side. The width of the main trunk of obturator nerve was 2.87 ± 0.64 mm over the right side and 2.82 ± 0.64 mm over the left side.

**Conclusions:**

We believe that morphometric data of the obturator nerve will be enlightening to the operating surgeon during procedures such as obturator nerve block, nerve transplantation, and obturator nerve repair. The dimensions of the obturator nerve observed in the present study can be utilized as a morphological database for our sample population.

## Introduction

Peripheral nerve injury is managed by exploration and nerve repair. Nerve regeneration is possible, but it has been reported to be associated with poor functional recovery. ^1^ The study of obturator nerve morphology may enlighten the clinician and provide successful clinical outcomes. Analysis of the morphology of the nerve may help the operating surgeon to assess the matching of the donor and recipient nerves. ^1^ Obturator nerve are formed in the lumbar plexus by the ventral division of the ventral rami of the L2, L3, and L4 nerves. It is also involved in several pathological processes. Obturator nerve entrapment can occur due to pressure injury around the obturator canal. ^2^ This contributes to pain and decreased sensation over the adductor compartment of the thigh. There may be difficulty in adduction of the thigh. ^3^ The iatrogenic causes of obturator neuropathy also include traumatic injury, iatrogenic injuries during the surgical procedures like hip arthroplasty, urological surgeries, and spine surgeries through the retroperitoneal approach. ^2^ The obturator nerve entrapment is observed in sports hernia and also in gynecological conditions like ectopic pregnancies. ^4^^–^^6^ The benign swellings like cysts, neurofibroma, and lipoma can also cause pressure effect over the obturator nerve at this location. ^7^^,^^8^ Due to all these implications, it is good to have the normal anatomical morphometric data of the obturator nerve with respect to that particular population. A literature search revealed that there was not much information available about this subject in the Indian sample population. This was the motivation for performing this anatomical investigation. The goal of this research was to study the morphology of the branching pattern of the obturator nerve and its morphometry in embalmed cadavers.

## Methods

In this study of the morphology of the human obturator nerve, we examined 50 embalmed cadavers. The sex category of the sample was not considered. Specimens showing pathological changes were not included in this anatomical study. A total of 100 obturator nerves were analyzed with respect to their topographical branching into the anterior and posterior divisions. The length, width, and thickness of the main trunk of the obturator nerve, anterior division, and posterior division were measured separately using Vernier calipers. Three measurements were taken by the same researcher of this study and the average of three was taken. This prevented the intra and inter-observer bias. The copyright license of the Vernier caliper is available in our department. Side-based comparisons were also performed using Student’s paired t-test. A recent version of the SPSS software (Version 27) was employed for statistical exploration. The copyright license of the SPSS software is available with our university. The topographical location of the obturator nerve and arrangement of structures at the obturator foramen were also examined. The mean distance of branching of the obturator nerve from the superior and inferior borders of the obturator foramen was measured. This anatomical study was approved (IEC KMC MLR 09-18/310) by the institutional ethics committee of Kasturba Medical College, Mangaluru, India (Reg. No. ECR/541/Inst/KA/2014/RR-17). This was approved from 26 ^th^ September, 2018. We state that this research adheres to the Declaration of Helsinki. The protocol of this study was archived in the dx.doi.org/10.17504/protocols.io.5qpvo317dv4o/v1.

## Results

**Figure 1.**
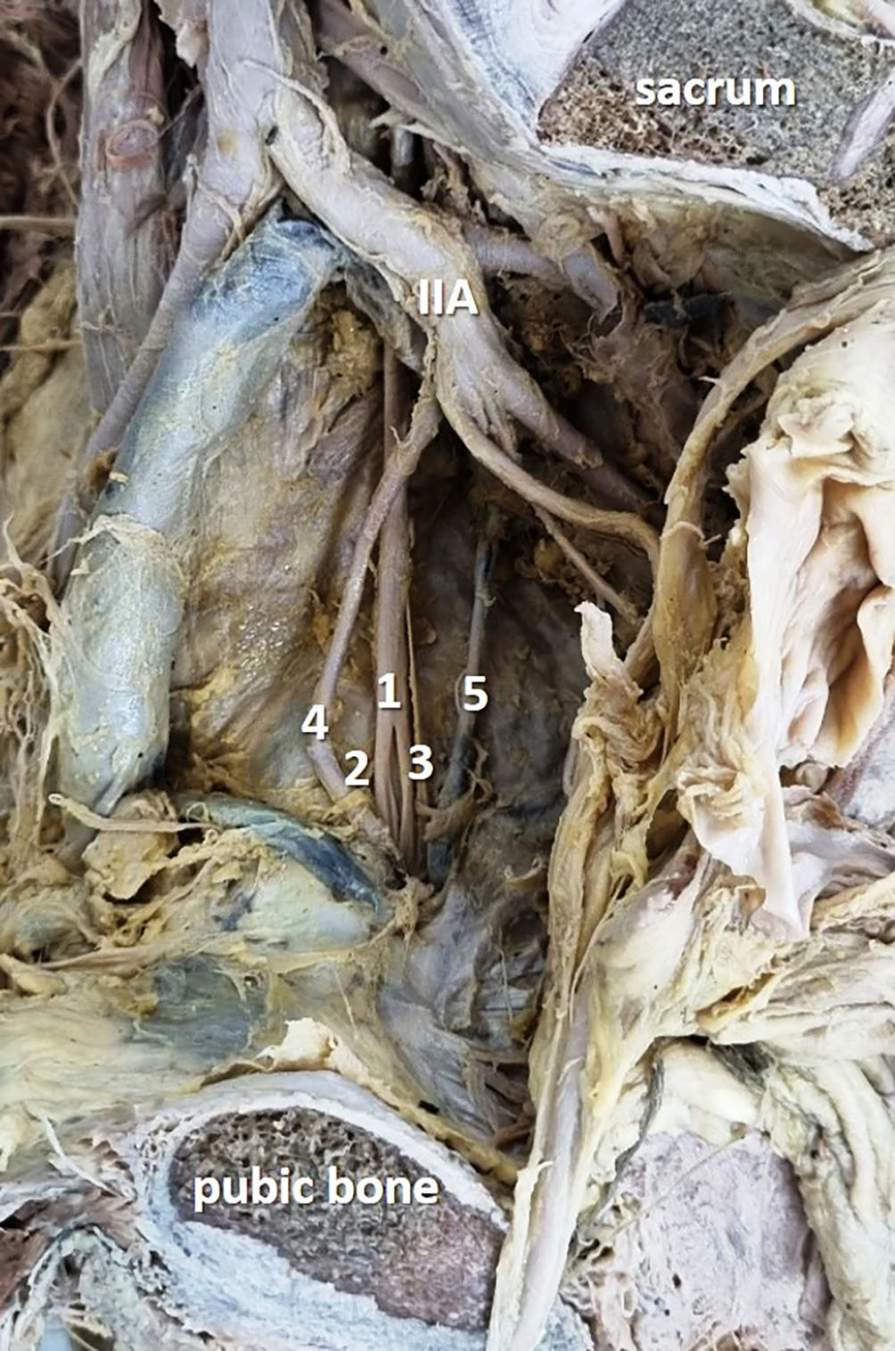
Embalmed cadaveric lower limb showing the intrapelvic division (56% cases) of the obturator nerve (1-obturator nerve; 2-anterior division; 3-posterior division; 4-obturator artery; 5-obturator vein; IIA-internal iliac artery).

**Figure 2.**
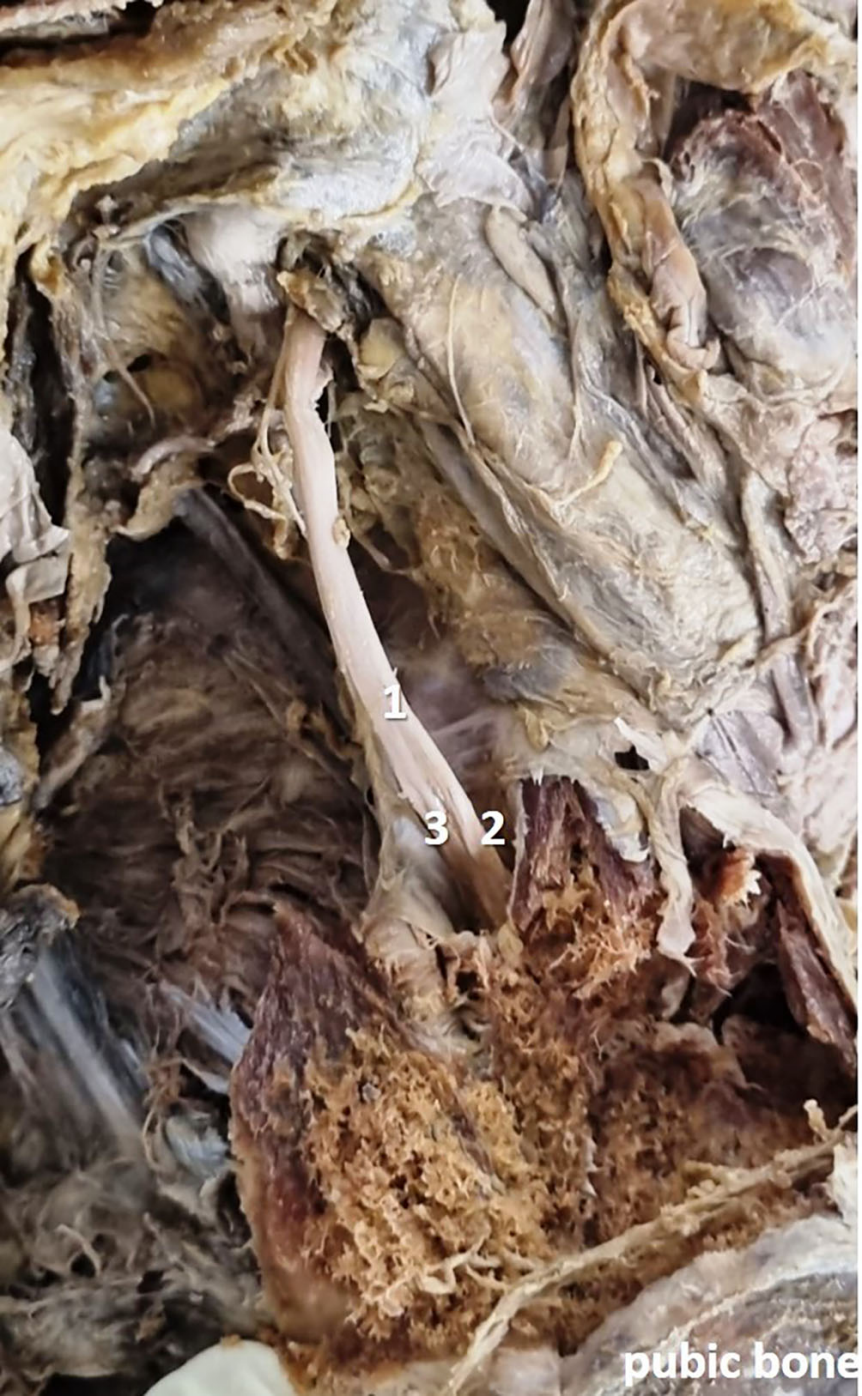
Embalmed cadaveric lower limb showing the branching of obturator nerve inside (24% cases) the obturator canal (1-obturator nerve; 2-anterior division; 3-posterior division).

**Figure 3.**
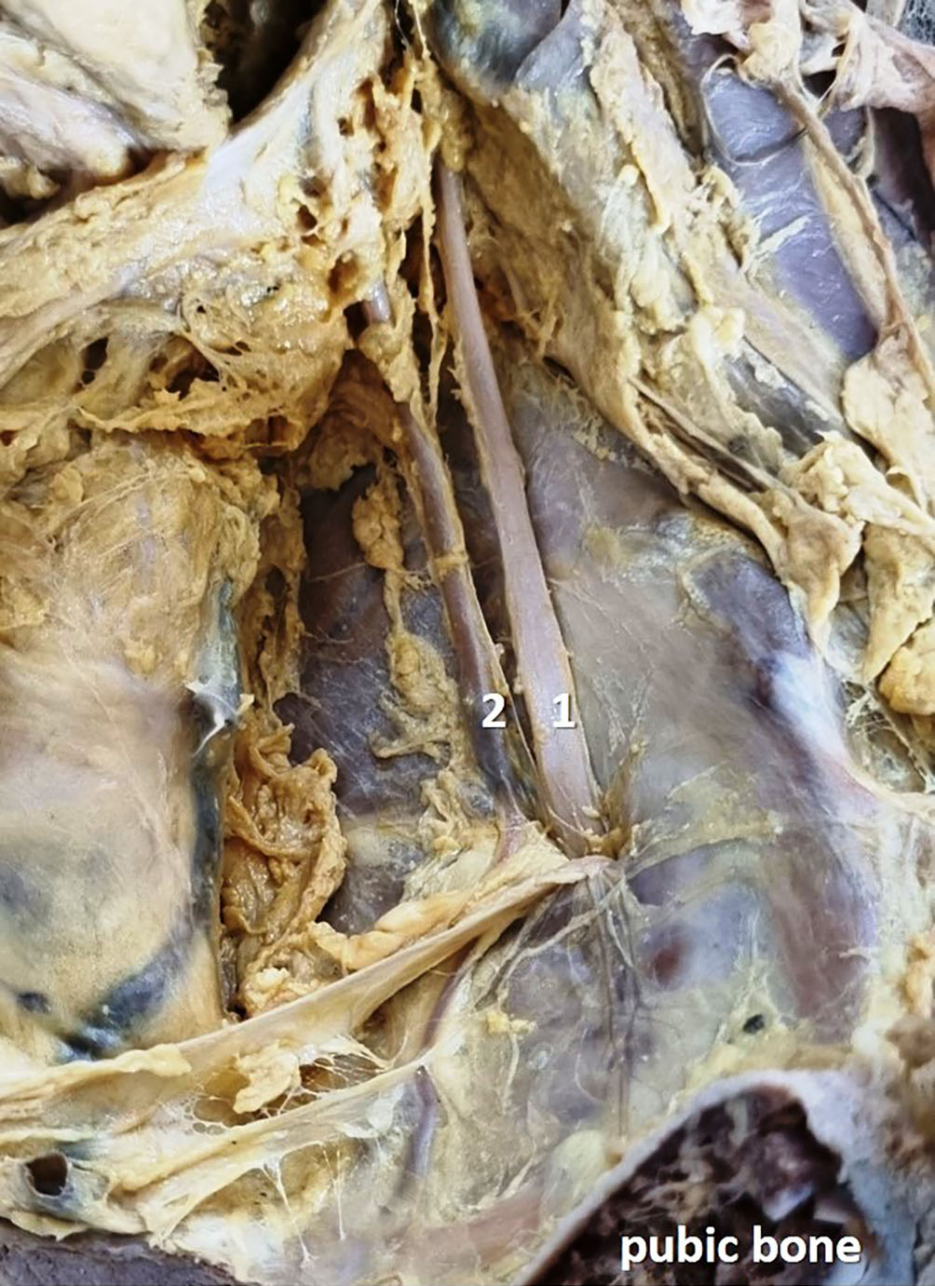
Embalmed cadaveric lower limb showing the undivided (20% cases) obturator nerve (1-obturator nerve; 2-obturator artery).

**Figure 4.**
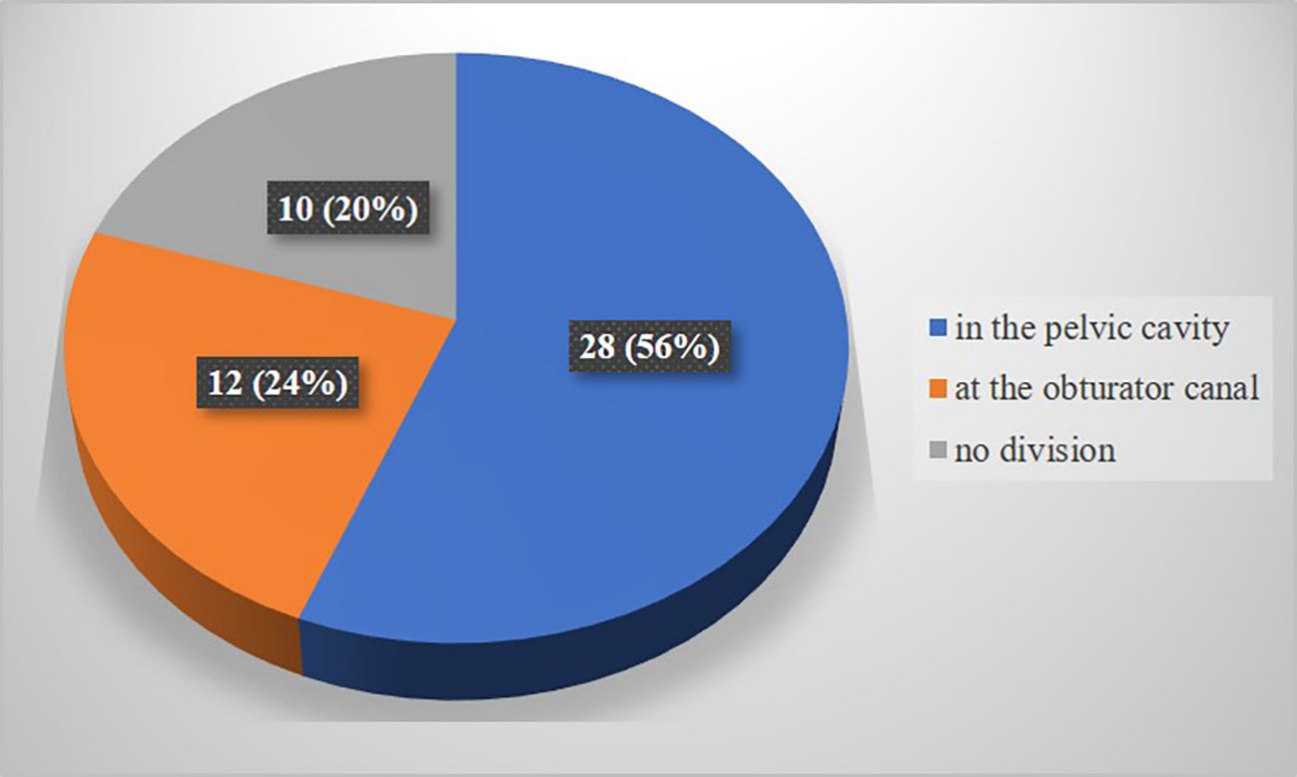
Frequency of topographic anatomy of division of obturator nerve (n=50).

In the present study, obturator nerve branching occurred at the pelvic cavity into the anterior and posterior divisions ( Figure 1) in 28 specimens (56%), at the obturator canal ( Figure 2) in 12 specimens (24%), and there was no division ( Figure 3) of the obturator nerve in 10 specimens (20%). The frequency of the topographic anatomy of the obturator nerve division is shown in Figure 4. The mean length, width and thickness of the trunk was 107.26 ± 8.71 mm, 2.84 ± 0.88 mm and 1.11 ± 0.35 mm individually. The mean width and thickness of the anterior and posterior divisions were 2.19 ± 0.82 mm, 0.9 ± 0.1 mm, 0.99 ± 0.6 mm and 0.71 ± 0.26 mm individually. The length of anterior and posterior division of obturator nerve were 109.01 ± 9.91 mm and 106.08 ± 7.71 mm respectively ( Table 1).

**Table 1.** Morphometric data of the obturator nerve (n=100).

Obturator nerve	Length	Width	Thickness
Trunk	107.26 ± 8.71 mm	2.84 ± 0.88 mm	1.11 ± 0.35 mm
Anterior division	109.01 ± 9.91 mm	2.19 ± 0.82 mm	0.9 ± 0.1 mm
Posterior division	106.08 ± 7.71 mm	0.99 ± 0.6 mm	0.71 ± 0.26 mm

The width of the main trunk was 2.87 ± 0.64 mm and 2.82 ± 0.64 mm over the right and left sides. The obturator nerve length was 107.55± 8.15 mm over the right side and 106.98± 9.27 mm at the left side. Its thickness measured 0.94 ± 0.07 mm and 1.25 ± 0.46 mm over the right and left sides. The length of anterior division was 110.88 ± 12.02 mm over the right side and 107.13 ± 7.81 mm over the left side. The width of the anterior division was 1.4 ± 0.55 mm and 1.7 ± 0.57 mm over the right and left sides. The thickness of anterior division was 0.94 ± 0.05 mm and 0.88 ± 0.13 mm over the right and left sides. The width of posterior division was 0.84 ± 0.21 mm and 1.06 ± 0.83 mm at the right and left sides. The thickness of posterior division was 0.54 ± 0.21 mm and 0.78 ± 0.29 mm at the right and left sides. The length of posterior division was 108.42 ± 9.31 mm and 105.54 ± 6.11 mm at the right and left sides ( Table 2). The topographical location of the branching of the obturator nerve from the upper and lower borders of obturator foramen was 1.48 ± 0.58 mm and 3.07 ± 1.1 mm.

**Table 2.** Side based comparison of morphometric data of the obturator nerve (n=50).

Obturator nerve	Parameter	Right	Left
Trunk	length	107.55 ± 8.15 mm	106.98 ± 9.27 mm
	width	2.87 ± 0.64 mm	2.82 ± 0.64 mm
	thickness	0.94 ± 0.07 mm	1.25 ± 0.46 mm
Anterior division	length	110.88 ± 12.02 mm	107.13 ± 7.81 mm
	width	1.4 ± 0.55 mm	1.7 ± 0.57 mm
	thickness	0.94 ± 0.05 mm	0.88 ± 0.13 mm
Posterior division	length	108.42 ± 9.31 mm	105.54 ± 6.11 mm
	width	0.84 ± 0.21 mm	1.06 ± 0.83 mm
	thickness	0.54 ± 0.21 mm	0.78 ± 0.29 mm

Statistical analysis, paired ‘t’ test, significance at p<0.05.

The difference was not statistically significant when the comparison was performed between the right and left sides for all parameters studied (p>0.05).

## Discussion

The clinical anatomy of obturator nerve is essential. The obturator nerve is related to the internal iliac artery and internal iliac vein at the lateral pelvic wall. It traverses the obturator canal and exits the pelvis. Its anterior and posterior divisions innervate muscles, eventually. The anterior division courses over the obturator externus muscle and the adductor brevis is located posterior to the anterior division of obturator nerve. ^9^ The pectineus and adductor longus muscles are found anterior to the obturator nerve. The other adductor compartment muscles, gracilis, adductor longus, and adductor brevis are also supplied by the obturator nerve. Itsposterior division pierces the obturator externus muscle and courses in between the adductor brevis and adductor magnus muscles. The muscular branches of obturator externus and adductor magnus muscles also offer articular twigs to the hip and knee joints. Its cutaneous branches supply the skin at the medial aspect of the thigh. ^9^ It was reported that, there exists significant anatomical variation in the branching and subdivisions of the obturator nerve. These variations cause difficulty in the accomplishing the regional anesthesia. ^10^ The obturator nerve division is known for its variations with respect to its topography in the obturator canal. ^11^ Berhanu et al. ^12^ observed intrapelvic division of obturator nerve in 23.9% cases, in the obturator canal in 44.8% cases and infrapelvic in 31.3% cases. In the present study, no extrapelvic divisions were observed (0%). In our study, the obturator nerve branched into the anterior and posterior divisions inside the pelvic cavity in 56% and 24% of the specimens, respectively. There was no division of the obturator nerve in 20% of samples. According to Tshabalala et al. ^11^ from the South African population, intrapelvic branching was observed in 2% of cases, and the majority were branching in the obturator canal (93%). Extrapelvic branching was observed in 5% of cases. In another study in Greece by Anagnostopoulou et al., ^10^ intrapelvic branching was observed in 25% of cases, branching within the obturator canal was observed in 23% of cases, and extrapelvic branching was observed in 52% of cases. The differences in the data from our study may be due to ancestral variations. Other than these two previous studies, data are not available regarding the dimensions of the obturator nerve. In this context, our anatomical research may help anatomists and anthropologists to compare these data.

The width of obturator nerve was 2.67 mm in males and 1.91 mm in females according to Yount et al. ^1^ In our study, the sex based comparison was not performed and the width was given for both the sexes together, which was measuring 2.84 ± 0.88 mm. Yount et al. ^1^ reported that the average width of obturator nerve over the left and right sides were 2.28 mm and 2.29 mm. In our study, these dimensions were2.82 ± 0.64 mm and 2.87 ± 0.64 mm. Yount et al. ^1^ also reported the length of obturator nerve, which was measuring 10.86 cm and 10.83 cm at the right andleft sides. Our data is almost similar to their data as the same dimensions in our study measured 107.55± 8.15 mm and 106.98± 9.27 mm. In this study, the distance of the branching of obturator nerve from the superior and inferior borders of obturator foramen were 1.48 ± 0.58 mm and 3.07 ± 1.1 mm.

The obturator nerve can be utilized as a nerve transfer for femoral nerve paralysis. ^13^ The neurectomy of anterior division of the obturator nerve and intrapelvic obturator neurotomy are performed to relieve spasticity in the medial compartment of the thigh in children suffering from cerebral palsy. ^14^ Lack of anatomical knowledge can lead to iatrogenic injury of the obturator nerve. Kendir et al. ^15^ emphasized the reputation of morphological awareness of the obturator nerve at the obturator canal in accomplishing the best obturator nerve block. Care should be taken while injecting the anesthetic drug simultaneously into the anterior and posterior divisions because of the variability in the branching of the obturator nerve. Anesthesia may be incomplete if there are variations in branching pattern. ^11^

This study was aimed at helping clinicians, but its limitations, such as individual branches to the muscles it supplies, were not explored. A gender-based comparison of morphometric data was not performed. This study did not record the prevalence of accessory obturator arteries. Future implications of this study include a more detailed topographical anatomy, such as the distance of the obturator nerve from the femoral artery. The distance between the anterior superior iliac spine and medial condyle of the femur can be determined to assess the ratio, and regression formulae could be obtained. The depth of the obturator nerve from the skin can also be determined. This may assist with the obturator nerve block procedure.

## Conclusion

The data of this study will be clinically helpful during the procedures like obturator nerve repair and transplantation. The dimensions of this study can be considered as the morphological database of Indian population.

## Ethics and consent

This anatomical study was approved (IEC KMC MLR 09-18/310) by the institutional ethics committee of Kasturba Medical College, Mangaluru, India (Reg. No. ECR/541/Inst/KA/2014/RR-17). This was approved from 26 ^th^ September, 2018. We state that this research adheres to the Declaration of Helsinki. The body donors of the cadavers, which are utilized in this study have consented to utilize their body for the medical research along with the medical teaching. They have put their signatures in their body donation form along with the witnesses.

## Data Availability

Figshare: Medline database search strategy for ‘Morphology of obturator nerve’. https://doi.org/10.6084/m9.figshare.24955383. ^16^ The project contains the following underlying data: file name – Obturator Nerve.xlsx The statistical analysis was performed by using the recent version of SPSS software. ^17^ Data are available under the terms of the Creative Commons Zero “No rights reserved” data waiver (CC0 1.0 Public domain dedication). Figshare: The strobe checklist. Anatomical study of obturator nerve. https://doi.org/10.6084/m9.figshare.25526056. ^18^ Data are available under the terms of the Creative Commons Zero “No rights reserved” data waiver (CC0 1.0 Public domain dedication).
