# Supplementary material for: Morphology of the obturator nerve, an anatomical study with emphasis on its clinical implications
Source: F1000Res. 2025 Mar 31;13:329. Originally published 2024 Apr 23. [Version 2] doi: 10.12688/f1000research.147000.2 (PMC12134726; doi:10.12688/f1000research.147000.2)
Supplement: Peer review history

# Reviewer report: Reviewer response for version 2

**DOI:** 10.5256/f1000research.179713.r385609

**Published:** 2025-06-03

**Authors:** Shubhangi Yadav

**Affiliations:** 1 All India Institute of Medical Sciences–Raebareli, Uttar Prades, India

Morphology of the obturator nerve, an anatomical study with emphasis on its clinical implications.

 This manuscript presents a well-structured, cadaveric cross-sectional study on the **morphological and morphometric characteristics** of the obturator nerve in an Indian population.

 Introduction: 

 This section effectively highlights the anatomical significance and clinical implications, particularly in the contexts of nerve blocks, pelvic surgeries, and nerve transfers. Additionally, the inclusion of reviewer-suggested content, such as a schematic diagram illustrating the measurements performed and the rationale behind these measurements, is a valuable enhancement that strengthens the overall clarity and relevance of the study.

 Methods

 The methods section is **clear, replicable**, and **meticulously detailed**. It demonstrates a robust approach to data collection with sufficient control for observer bias.  However, the inclusion of a sex-based analysis would have further strengthened the study.

 Results

 The results are presented systematically with relevant **tables and figures**, making it easy to interpret the data. The clear delineation of measurements and side-based comparison is informative and relevant for surgical applications.

 Discussion 

- The comparison with studies from different populations (e.g., Greece, South Africa) underscores **geographical anatomical variation** and highlights the need for **region-specific data**.
- Emphasis on applications in **nerve block**, **spasticity relief**, and **nerve transfer** is clinically valuable.
- Acknowledging anatomical variability enhances the practical importance of the research.
- The limitations are **openly discussed**, such as the lack of sex-based analysis and missing data on accessory branches, indicating the authors' **transparency** and scientific rigor.
- Suggestions for **future studies** are well-thought-out and relevant.

Conclusion 

 The conclusion reinforces the practical application of the morphometric findings and reiterates the study’s importance in clinical settings. The emphasis on laparoscopic and nerve transfer procedures adds significant **clinical value** to the anatomical findings.

Is the work clearly and accurately presented and does it cite the current literature?

Yes

If applicable, is the statistical analysis and its interpretation appropriate?

I cannot comment. A qualified statistician is required.

Are all the source data underlying the results available to ensure full reproducibility?

Yes

Is the study design appropriate and is the work technically sound?

Partly

Are the conclusions drawn adequately supported by the results?

Yes

Are sufficient details of methods and analysis provided to allow replication by others?

Yes

Reviewer Expertise:

Anthropometry, Embryology, Teratology, Medical Education

I confirm that I have read this submission and believe that I have an appropriate level of expertise to confirm that it is of an acceptable scientific standard.

# Reviewer report: Reviewer response for version 2

**DOI:** 10.5256/f1000research.179713.r385604

**Published:** 2025-05-27

**Authors:** Marc-Olivier St-pierre

**Affiliations:** 1 Génie des systèmes, Ecole de technologie superieure, Montreal, Québec, Canada

Report F1000 research

**Morphology of the obturator nerve, an anatomical study with emphasis on its clinical implications**

 The article presented by the authors reveals the morphological characteristics of the obturator nerve as well as its various divisions. The subject of the article is interesting, but I believe several aspects need to be revised to improve the delivery of the message. Work needs to be done on the introduction and discussion sections. Additionally, I believe that a comparison between men and women is essential. It should not be necessary to write another article to address these differences. If the authors have the sex of each evaluated specimen, I believe that adding a comparison between sexes does not represent a significant amount of work. This last aspect is crucial for the acceptance of this paper.

 I believe the English should be reviewed by an external party, or at the very least, a thorough proofreading by the authors is necessary.

**Comments for each specific section**

Abstract

- I don't believe the use of the term "trunk" is necessary. I would only use "obturator nerve."
- The length, width and thickness of the trunk of obturator nerve was were 108.26 ± 9.53 mm, 2.84 ± 0.88 mm and 1.11 ± 0.35 mm, respectively.
- The width and thickness of the anterior and posterior divisions of obturator nerve measured 2.19 ± 0.82 mm, 0.9 ± 0.1 mm, 0.99 ± 0.6 mm and 0.71 ± 0.26 mm.
  - I would separate the anterior and posterior in two sentences. It would increase clarity.
- Check digits after decimal point (one or two) please revise in the manuscript.
- Please revise the results section.
  - First describe all measurement for the obturator nerve. After, describe all measurements for the anterior and posterior divisions.

Introduction

 Introduction should be revised. Here are some tips that could help (I think).

- Organize the idea as follow:
  - Obturator nerve function
  - Obturator implication in clinical problem
  - Treatment technique and here reports the problem (variability for nerve block)
  - State the literature on obturator nerve variability.
  - State the importance of the research (third and fourth sentences)
  - State objectives
- I believe all the information is currently in the introduction. However, it is presented somewhat randomly.

More specific comments:

- Don’t need this sentence: *A literature search revealed that there was not much information available about this subject in the Indian sample population*

Methods

- After the first sentence, state the ethical information.
- **Why not compare the sex differences. This is a main concern. **
- Define: pathological changes.
- Vernier calipers: report brand, company, country, precision.
- Can you show point A and B on figure 2? Do you have any model in your lab that could be used to replace figure 1?  
- Please *revise: *The branch's point of origin to the adductor longus and adductor magnus muscles were considered as the distal points for the length of anterior and posterior divisions,* respectively*. What do you mean?
- Have you measured the rater repeatability on two or three specimens? Follow-up question: how many raters were taking the measurements?
- No need to report this: *The copyright license of the SPSS software is available with our university.*
- This should be described beforehand: *The mean distance of branching of the obturator nerve from the superior and inferior borders of the obturator foramen was also measured. *You should also describe this measure in the figure.
- Have you stated how you define nerve branching? Maybe make it more obvious for the reader because its your first result.

Results

- Table 1: see decimals
- Tables 1 and 2: You can indicate the unit of measurement in the table title, which will simplify the tables.
- I understand that the comparison between the left and right sides shows no significant difference. However, have you conducted tests to distinguish morphometric differences between the anterior and posterior branches?

Discussion

- This should be the second sentence of your discussion: *It was reported that, there exists significant anatomical variation in the branching and subdivisions of the obturator nerve. These variations cause difficulty in the accomplishing the regional anesthesia. The obturator nerve division is known for its variations with respect to its topography in the obturator canal. *Everything before this can be erase.
- *In the present study, no extrapelvic divisions were observed (0%). *Why include this? You do not report any extrapelvic division for the study by Berhanu et al. I believe you can remove it.
- I believe you should describe all the studies that report the position of the division and then present your results. By reporting your results in the middle, it creates a break in the flow of these results.
- *Other than these two previous studies, data is not available regarding the dimensions of the obturator nerve. *Which studies?
- *In this context, our anatomical research may help anatomists and anthropologists to compare these data.* You could replace it with: Although these studies report the position of the obturator nerve division, none of them present the morphological data of the nerve and its divisions. And begin the next paragraph with: To the best of our research, the study by Young et al. is the only group of authors that has addressed this topic.
- **I believe it is crucial to analyze the results by comparing sexes. The study by Young et al. shows a significant difference of nearly 0.7 mm between men and women. This difference suggests that clinical procedures could be more complex in women due to their smaller volume. In this regard, your distinction between the anterior and posterior branches, with the posterior appearing smaller, adds to this. Would nerve blocking of the obturator nerve (posterior branch) be too risky given its small diameter?**

Conclusion

- The first sentence of the conclusion is way to long. Please revise.

Figures

- Add axis: proximal, distal, medial and lateral to help understand close view in figures.

Is the work clearly and accurately presented and does it cite the current literature?

Partly

If applicable, is the statistical analysis and its interpretation appropriate?

Partly

Are all the source data underlying the results available to ensure full reproducibility?

Yes

Is the study design appropriate and is the work technically sound?

Partly

Are the conclusions drawn adequately supported by the results?

Yes

Are sufficient details of methods and analysis provided to allow replication by others?

Yes

Reviewer Expertise:

Human anatomy, biomechanics, cadaveric studies

I confirm that I have read this submission and believe that I have an appropriate level of expertise to confirm that it is of an acceptable scientific standard, however I have significant reservations, as outlined above.

# Reviewer report: Reviewer response for version 1

**DOI:** 10.5256/f1000research.161133.r279366

**Published:** 2024-05-29

**Authors:** Najma Mobin

**Affiliations:** 1 JSS Academy of Higher Education & Research, Mysore, Karnataka, India

The introduction is well written. The methodology and results are explained well. The pictures are self- explanatory.  The implications in discussion are well written and explained. Limitations of the study are mentioned. Conclusion requires minor changes, like in what way this study is going to help in nerve repair and transplant.

Is the work clearly and accurately presented and does it cite the current literature?

Yes

If applicable, is the statistical analysis and its interpretation appropriate?

I cannot comment. A qualified statistician is required.

Are all the source data underlying the results available to ensure full reproducibility?

Yes

Is the study design appropriate and is the work technically sound?

Yes

Are the conclusions drawn adequately supported by the results?

Partly

Are sufficient details of methods and analysis provided to allow replication by others?

Yes

Reviewer Expertise:

Plastination, museum techniques, histological and gross anatomy studies.

I confirm that I have read this submission and believe that I have an appropriate level of expertise to confirm that it is of an acceptable scientific standard.

# Response

**Published:** 2024-06-30

**Authors:** B.V. Murlimanju

**Affiliations:** ANATOMY, Manipal Academy of Higher Education, Mangalore, Karnataka, India

We thank the learned reviewer for the expert opinion. The conclusion of this study is revised as per the suggestion. Some of the nerve repair and transplantation surgeries are performed laparoscopically. The prior knowledge of morphological dimensions and branching pattern will assist the surgeons in easy access of the obturator nerve during these procedures.

# Reviewer report: Reviewer response for version 1

**DOI:** 10.5256/f1000research.161133.r273599

**Published:** 2024-05-10

**Authors:** Rajasekhar SSSN

**Affiliations:** 1 Jawaharlal Institute of Postgraduate Medical Education and Research, Puducherry, India

The study describes the measurement of obturator morphometric parameters and collation of data without gender differentiation. 

 discussion section: the hypothesis given for the difference between the results of the current study and that of the another study done in Greece is not a valid one " Ancestral variations"

 Comments:  

 Schematic diagram representing the measurements can be provided.

 Rationale for the study including the rationale for selecting the types of measurements should be given. 

 There is a specimen number discrepancy between the abstract and the methods section. 

 Methods section mentions about the copyright licence for Vernier callipers, please clarify this. 

 The term "gender" should be used instead of "sex". 

 Justify the rationale for the measurement of width of obturator nerve. 

 Topographical measurement parameters were inconsistent between the methods and the results: obturator nerve division versus obturator nerve. 

 Specify which point is taken as distal point for the measurement of length of both anterior and posterior divisaion of the nerve. 

 Gender differentiation is not done, but the measurements such as length and width may be significantly different/smaller in females compared to males, owing to the shorter stature of females compared to males.

 Therefore the combined data that includes males and females cannot be considered as a valid data to apply or generalise the data for the wider population.

 Consequently, the results of the study may not be valid. The authors need to review the methods used in the study and rework the manuscript with results worked out separately for males and females to obtain meaningful information from the study.

Is the work clearly and accurately presented and does it cite the current literature?

Partly

If applicable, is the statistical analysis and its interpretation appropriate?

Yes

Are all the source data underlying the results available to ensure full reproducibility?

Yes

Is the study design appropriate and is the work technically sound?

Yes

Are the conclusions drawn adequately supported by the results?

No

Are sufficient details of methods and analysis provided to allow replication by others?

No

Reviewer Expertise:

Areas of expertise include gross anatomy, clinical anatomy, embalming, histological techniques, and histology.

I confirm that I have read this submission and believe that I have an appropriate level of expertise to state that I do not consider it to be of an acceptable scientific standard, for reasons outlined above.

# Response

**Published:** 2025-03-20

**Authors:** B.V. Murlimanju

**Affiliations:** ANATOMY, Manipal Academy of Higher Education, Mangalore, Karnataka, India

Dear Sir,

 We have made the changes per your suggestion. 

 A schematic diagram with labelling representing the measurements performed in this study is added in this revised version along with the detailed figure legend. The rationale of the study for performing the types of measurements including the rationale for measuring the width are added in the introduction section. Since the reviewer did not accept our hypothesis, ancestral variations which was given for the difference between the results of the current study and that of another study done in Greece, this was deleted. The specimen number discrepancy between the abstract and the methods section, cadaver Vs. cadaveric lower limb is corrected. The sentence mentioning the copyright license for Vernier calipers is deleted. The term ‘gender’ is replaced with the word ‘sex’. The discrepancy of topographical measurement parameters between the methods and the results, obturator nerve division versus obturator nerve has been corrected and revised. The distal point for the measurement of length of both anterior division (till the origin of muscular branch to the adductor longus) and posterior division (till the origin of muscular branch to the adductor magnus) of the nerve are added as per the reviewer opinion. The conclusion was revised by adding the rationale of performing this study to assist in the nerve repair, nerve block and transplant procedures.
